# Active Early: one-year policy intervention to increase physical activity among early care and education programs in Wisconsin

**DOI:** 10.1186/s12889-016-3198-3

**Published:** 2016-07-20

**Authors:** Tara L. LaRowe, Emily J. Tomayko, Amy M. Meinen, Jill Hoiting, Courtney Saxler, Bridget Cullen, the Wisconsin Early Childhood Obesity Prevention Initiative (WECOPI)

**Affiliations:** Department of Dietetics, School of Natural and Health Sciences, Mount Mary University, 2900 North Menomonee River Parkway, Milwaukee, WI 53222 USA; College of Public Health & Human Sciences, School of Biological & Population Health Sciences, Oregon State University, 118D Milam Hall, Corvallis, OR 97331 USA; Department of Family Medicine, School of Medicine and Public Health, University of Wisconsin-Madison, 610 Walnut Street, Madison, WI 53726 USA; Supporting Families Together Association, 700 Rayovac Drive, Suite 6, Madison, WI 53711 USA; School of Medicine and Public Health, Area Health Education Centers System, University of Wisconsin-Madison, 750 Highland Avenue, Madison, WI 53705 USA; State of Wisconsin, Department of Children and Families, 201 East Washington Avenue, Madison, WI 53708 USA

**Keywords:** Obesity prevention, Early care and education, Physical activity, Accelerometry, Environment and policy assessment and observation

## Abstract

**Background:**

Early childcare and education (ECE) is a prime setting for obesity prevention and the establishment of healthy behaviors. The objective of this quasi-experimental study was to examine the efficacy of the Active Early guide, which includes evidenced-based approaches, provider resources, and training, to improve physical activity opportunities through structured (i.e. teacher-led) activity and environmental changes thereby increasing physical activity among children, ages 2–5 years, in the ECE setting.

**Methods:**

Twenty ECE programs in Wisconsin, 7 family and 13 group, were included. An 80-page guide, Active Early, was developed by experts and statewide partners in the fields of ECE, public health, and physical activity and was revised by ECE providers prior to implementation. Over 12 months, ECE programs received on-site training and technical assistance to implement the strategies and resources provided in the Active Early guide. Main outcome measures included observed minutes of teacher-led physical activity, physical activity environment measured by the Environment and Policy Assessment and Observation (EPAO) instrument, and child physical activity levels via accelerometry. All measures were collected at baseline, 6 months, and 12 months and were analyzed for changes over time.

**Results:**

Observed teacher-led physical activity significantly increased from 30.9 ± 22.7 min at baseline to 82.3 ± 41.3 min at 12 months. The change in percent time children spent in sedentary activity decreased significantly after 12 months (−4.4 ± 14.2 % time, −29.2 ± 2.6 min, *p* < 0.02). Additionally, as teacher led-activity increased, percent time children were sedentary decreased (*r* = −0.37, *p* < 0.05) and percent time spent in light physical activity increased (*r* = 0.35, *p* < 0.05). Among all ECE programs, the physical activity environment improved significantly as indicated by multiple sub-scales of the EPAO; scores showing the greatest increases were the Training and Education (14.5 ± 6.5 at 12-months vs. 2.4 ± 3.8 at baseline, *p* < 0.01) and Physical Activity Policy (18.6 ± 4.6 at 12-months vs. 2.0 ± 4.1 at baseline, *p* < 0.01).

**Conclusions:**

Active Early promoted improvements in providing structured (i.e. teacher-led) physical activity beyond the recommended 60 daily minutes using low- to no-cost strategies along with training and environmental changes. Furthermore, it was observed that Active Early positively impacted child physical activity levels by the end of the intervention. However, resources, training, and technical assistance may be necessary for ECE programs to be successful beyond the use of the Active Early guide. Implementing local-level physical activity policies combined with support from local and statewide partners has the potential to influence higher standards for regulated ECE programs.

## Background

Childhood obesity is a public health priority in both the United States and in the state of Wisconsin (WI). A recent report from the Centers for Disease Control and Prevention suggests that while many states recently experienced decreases in early childhood obesity rates, Wisconsin experienced a slight increase, with nearly 30 % of low-income 2–4 year olds categorized as overweight or obese [1]. Moreover, obesity by age 5 has been shown to be associated with a four-fold increase in obesity risk in adolescence [2], which is subsequently predictive of adult obesity and other chronic disease risk [3, 4]. Early childhood is clearly a vital window for developing health-related habits to prevent obesity. One such health-related behavior that has been identified as a key prevention target for obesity prevention is physical activity [5].

Physical activity in children has been associated with improved physical, behavioral, cognitive, and social outcomes [6, 7]. Moreover, previous research has suggested preschool-age children may be more receptive to diet and physical activity related behavior change compared to older children [8].

Despite the recognition of the importance of this early life period in child development across multiple domains, relatively few obesity prevention studies targeting preschool-age children have been conducted, with most research and public health messaging efforts directed toward school age children [9]. During early childhood, physical activity among children is under the influence of caregivers, including family members and early childcare and education (ECE) providers. In the state of WI, approximately 70 % of children ages 2–5 years spend time in some type of ECE program, often for the majority of the day [10]. In 2012, there were 4,549 licensed ECE facilities identified, serving a reported 228,744 children [10]. These ECE settings are therefore critical venues to impact health and prevent excessive weight gain [5], through strategies such as obtaining adequate physical activity levels.

Preschool aged children are not currently meeting minimum recommendations for physical activity [11–13]. In 2011, the Institute of Medicine recommended 15 min of physical activity per hour in care, which translates to 120 min per 8-h day [14]. Other public health agencies, both domestic and international, recommend similar levels of at least 90 to 120 min per day of a combination of unstructured and structured (i.e. teacher-led) daily activity for this age group. Current licensing standards in Wisconsin and many other states do not specify a mandated level of physical activity for regulated child care programs. Wisconsin’s quality improvement rating system for ECE, YoungStar, includes “at least 60 min [teacher-led] of daily physical activity” as one of four health and well-being quality indicators [15].

In Wisconsin, data suggest more than half of older children do not achieve even 60 min of daily physical activity during the course of their day [16], and information on adherence to activity recommendations in younger children is lacking [17]. Specifically, within the ECE setting, adherence may be low due to a variety of reasons, including lack of training and inadequate resources [18].

The objective of the quasi-experimental Active Early intervention was to increase physical activity among children, aged 2–5 years, in Wisconsin by promoting evidence-based approaches for improving structured (i.e. teacher-led) and unstructured activity. Active Early recommends at least 120 min of structured and unstructured physical activity daily, with at least 60 min being structured (i.e. teacher-led). We hypothesized that ECE programs participating in the intervention would: 1) increase the amount of physical activity offered to children, specifically structured activity, (i.e.teacher-led) and 2) improve the physical environment and policies to support physical activity, thereby 3) increasing the amount of physical activity among children. Furthermore, we sought to understand whether intervention effects differed between the ECE programs that had more physical activity resources and supports at baseline versus those that had lower resources and supports.

## Methods

The Active Early program was part of a larger statewide program, funded through the Center for Disease Control and Prevention’s Communities Putting Prevention to Work (CPPW), that focused exclusively on increasing physical activity in the ECE setting. The general purposes of the CPPW grants were to identify health problems and improve services and benefits exclusively for those communities participating in the grant. The Wisconsin Department of Health Services’ Nutrition, Physical Activity, and Obesity Program (NPAO) was awarded the CPPW grant. The project design, guide, trainings to support the guide, and evaluation were developed from partners of the statewide coalition, the Wisconsin Early Childhood Obesity Prevention Initiative (WECOPI). WECOPI partners include NPAO, Supporting Families Together Association, Wisconsin Child Care Resource and Referral Agencies, Wisconsin Early Childhood Association, University of Wisconsin-Madison Department of Family Medicine, Wisconsin Council of Children and Families, Wisconsin Department of Children and Families, Wisconsin Department of Public Instruction, and other key stakeholders.

### Active early guide development

Prior to developing the Active Early guide, WECOPI members conducted formative assessments of the Wisconsin ECE setting, including an extensive literature review, ECE provider focus groups, parent focus groups, and key informant interviews with relevant local, state, and regional organizations. Key themes that emerged from these data sources were a lack of understanding of physical activity requirements and inadequate training and resources to achieve physical activity recommendations. Results from these local data were used to develop an 80-page guide (*Active Early: A Wisconsin guide for improving childhood physical activity* [19]*, available in English and Spanish*) that provided user-friendly tools and ideas while incorporating current science, public health research, and national recommendations. The guide provides an overview and addresses current research, public health practice, and national recommendations around 6 key areas related to physical activity: Development, Child Assessment, Routines, Environment, Resources, and Business Practices. Table 1 provides a summary of the content within each of the six key areas of the Active Early guide. Sample daily routines, activity ideas, equipment, and materials were included to promote the increased structured physical activity. The Guide also included examples of addressing cultural competence and inclusive approaches within each key areas. For example, providers are encouraged to understand daily home routines, such as meal times and family activities, as this reflects family values and priorities. Additionally, providers may need to modify activities and routines to accommodate children with physical disabilities or developmental delays. Beyond the center-focused guidance, each area includes information on engaging both families and communities around increasing physical activity. Throughout the guide’s development, revisions were made based ECE provider feedback.

**Table 1:** Key components in active early: a Wisconsin guide for improving childhood physical activity

Component	Content summary	Example activities and tips
1. Development	• Physical development o Gross motor skills o Fine motor skills • Brain development • Language development	Animal Movements: children move like their favorite animal while music is playing (e.g. walk like a crab or hop like a bunny) Tip: talk about movements using vocabulary that will help children understand their activities
2. Child Assessment	• Tools to assess child development • Documentation of child assessments • Action steps	Follow the Leader: have older children lead different activities so caregivers can observe the skills of younger children Tip: have an older child teach a new physical activity or gross motor skill to a younger child
3. Daily Routines	• Schedules • Transition times • Lesson planning	Active Clean Up: during clean up time, have children use a different traveling skill, such as walking fast, hopping, or jumping, as they put away materials Tip: try a few teacher-led physical activities at the end of outdoor play as a way to make transitions smoother
4. Environment	• Indoor space • Outdoor space	Balancing: explore balancing at both high and low levels. High-level positions include standing on tip-toe, on tip-toes with both feet and knees bent; on tip-toes with eyes closed. Low-level positions include balancing on two hands and one knee, one hand and two knees, etc. Tip: make dramatic play more active by providing materials for movement such as scarves or have children act out being an aerobics or yoga instructor
5. Resources	• Physical activity curriculums • Equipment • Materials • Books, websites • Assessment tools	Activity: research the Active Early Guide suggested physical activity curriculums; make a list of equipment and materials needed, etc. Tip: to minimize costs, use resources such as public libraries or state Child Care Information Centers
6. Business Practices	• Policy definitions • Types of policies • Policy development	Policies can help to: • Create consistent messages for staff, parents, and licensing officials • Provide clear guidelines for staff members and families • Provide basis for evaluation of program and identify areas for improvements Tip: policies set the stage for best practices; but remember that a policy is only as good as its implementation!

Active Early: A Wisconsin guide for improving childhood physical activity [https://www.dhs.wisconsin.gov/publications/p0/p00280.pdf]

### Site selection

Twenty regulated Wisconsin programs that served children aged 2–5 years old, had been in business for at least 3 years, and participated in the Child and Adult Care Food Program were selected by project partners to participate in the evaluation of Active Early. In total, 7 family providers, 7 small group centers (i.e., licensing capacity of ≤50), and 6 large group centers (i.e., licensing capacity ≥51) were included.

### Intervention

Participating child care centers received the Active Early intervention over 12 months. Participating sites received an initial 4-h training (Wisconsin Early Care and Education Physical Activity Training) that aligned with the Active Early guide. Delivery of the training utilized the existing structure of ECE training and technical assistance provided by three Wisconsin organizations (The Wisconsin Early Childhood Association, the Wisconsin Council on Children and Families, and Supporting Families Together Association). The objectives of this training were to provide background information regarding obesity prevention through physical activity programming and to demonstrate how to utilize the Active Early guide. One hundred and thirty-four ECE providers from the 20 intervention sites attended the 4-h in-person training. After the initial 4-h training, an additional 1-h follow-up in-person session was provided. Additionally, participating centers were provided with on-going unlimited technical assistance from trained consultants around the Active Early guide throughout the intervention upon their request. Technical assistance was provided in the modes of onsite, phone, and email assistance. Project-related training and technical assistance were designed to operate within existing infrastructure for these types of activities. In addition, each site received a micro-grant at the beginning of the intervention designed to facilitate sustainable investments in physical activity and related behaviors ($2,500 for family providers, $5,000 for small group centers, $7,500 for large group centers). The Active Early intervention was reviewed by the University of Wisconsin-Madison’s Institutional Review Board and was granted exemption from full review because they considered this project to be evaluation of a public service program. All staff participated in the deidentified evaluation unless verbally declining and all children participated in deidentified evaluation activities unless their caregivers submitted an opt out form.

### Data collection

Main outcome measures included site physical activity environment, child physical activity levels, and structured activity, (i.e. teacher-led activity). Except where noted, outcome data were collected at baseline, 6 months (midpoint), and 12 months (final) of the intervention by trained researchers:

#### Physical activity self-assessment (baseline only)

Each site completed a modified version of the Nutrition and Physical Activity Self-Assessment for Child Care (NAP SACC) [20] instrument which included evaluation around the following areas: active/inactive play time, play environment, physical activity, and physical activity policy. Results were used to direct each site in developing an action plan to achieve the intervention goals.

#### Site demographics (baseline only)

Information on the site, staff, child demographics, parent engagement, perceived barriers to implementing physical activity recommendations, and training needs were collected from the director of each center by the project assistant during phone or in-person interviews or via questionnaire.

#### Physical activity observations

Child care physical activity environment and policies were assessed using the Environment and Policy Assessment and Observation (EPAO) instrument, which involves a day-long observation, by trained staff, of the nutrition and physical activity environment of the child care program as well as a document review of program policies, activities, and food served (e.g. menu, parent handbook). Day-long observations started when then majority of children congregated in the morning until they were picked up by their guardians at the end of the day (typically an eight- to ten- hour day). Total physical activity environment was calculated from the EPAO using eight constructs, or subscales, that identify key physical activity indicators [20, 21]: Active Opportunities (e.g. total time active play was observed), Sedentary Opportunities (e.g. total minutes of seated activity observed), Portable Play Equipment (e.g. presence of play equipment), Fixed Play Equipment (e.g. equipment and space that is fixed within center area), Sedentary Environment (e.g. items that promote or discourage physical activity behavior), Staff Behaviors (e.g. interactions between staff and children that may promote or restrict active play), Physical Activity Training and Education (e.g. training and education for children, staff and parents), and Physical Activity Policy (e.g. general strength and content of physical activity policies). The eight subscales consisted of 75-item responses that observers rated and converted to a 3-point scale (0, 1 and 2). Scores were averaged within each subscale, and multiplied by 10, for a range of 0 to 20 (higher scores reflecting a more supportive environments). The total physical activity score represents the average of all subscale scores, again, ranging from 0 to 20 with higher scores reflecting more supportive environments. Detailed scoring of the EPAO has been previously published [21]. In addition to the EPAO protocol being administered at each observation, staff recorded the number of structured (i.e. teacher-led) physical activity occasions to quantify minutes of teacher-led physical activity. For larger group centers that had age-specific classrooms, the 3-year old classroom was chosen for observation to increase the number of children who would still be enrolled at the 12-month observation point, where they were observed in the 4-year old classroom.

#### Child physical activity

Child physical activity (PA) was measured using Actical triaxial accelerometers (Bio-Lynx, Montreal, Quebec, Canada). Accelerometers were attached to an adjustable belt worn by the children on the hip for an entire observation day (mean ± SD minutes worn at baseline was 420.9 ± 114.6 min). The accelerometers provided activity counts for each 15-s interval. Data were uploaded into the Actical 2.0 program for analysis. Data were reduced to quantify activity into counts/minute intervals and further to quantify the number of intervals for sedentary, light, and combined moderate plus vigorous physical activity (MVPA) per hour. Previous research has established accelerometer activity count thresholds for physical activity intensity categories. Puyau et al. [22] had children wearing Actical accelerometers perform preset activities while in a respiratory room calorimeter to determine cutoffs for sedentary (<100 cpm), light (≥100 < 1500 cpm), MVPA (≥1500 cpm). Although activity levels for children lack defined categories [23, 24], we chose these cutoffs because they represent typical physical activity behaviors for this age group of children. We used these cut points to calculate the percent time the children in our study spent wearing the accelerometers in each intensity category. We chose to analyze the data primarily in ‘percent time’ over total minutes because the total amount of time spent in child care varied per child. We then back-calculated total minutes from percent time for better interpretation of the data.

### Statistical analysis

Analyses with quantitative measures were performed with SAS software version 9.2 (SAS Inc., Cary, NC). Outcome variables related to the childcare physical activity environment and policies, structured activity (i.e. teacher-led activity), and child physical activity levels were compared before and after the 12-month intervention. To assess whether differences existed between sites that had high better supportive physical activity environments at baseline compared to low supportive environment centers, analyses were further stratified by PA-EPAO scores at baseline above and below the median score (score of 11.2). The median was used because of the small sample size and because established cut-points for high and low PA-EPAO have not been established. Results were not significantly different among group versus family sites; therefore, these data will not be reported. Differences in outcome measures used various statistical tests as appropriate, including mean differences *t*-tests, matched pairs *t*- tests, Pearson correlations, general linear models (PROC GLM) with fixed and random effects, and Tukey’s test of multiple comparisons. An alpha level of 0.5 was set for all significance testing.

## Results

### Site characteristics

The reach of the intervention included approximately 223 staff and 456 children from 13 rural and 7 urban sites; however, not all staff and children at each site participated in the evaluation. Reasons for not participating included: not being present at the time of evaluation, not having enough accelerometers for each child in the center, or staff and children electing not to participate. Table 2 reflects characteristics, reported by ECE directors, of those participating in the evaluation. The largest proportion of teachers and staff, reported from program directors, were 26–40 years old (43.3 %) and had some college education (52.8 %). The average enrollment of 2–5 year old children was 22.9 ± 19.1 and the estimated racial/ethnic distribution was 73.3 % white/Caucasian, 8.9 % African American, 7.6 % Hispanic, 8.0 % American Indian, and 2.1 % multiple race/ethnicities (Table 2). All sites fully completed the study protocol and associated testing. Total hours of technical assistance, from baseline to 12-monhts, was approximately 657 h for onsite and 298.5 h for offsite (each center and group providers received approximately 35 onsite and 14 offsite hours and each family centers received approximately 29 onsite and 17 offsite hours).

**Table 2:** Active Early ECE program demographics and characteristics

	All programs (*n* = 20)
Type of Program, *n*	
Group	13
Family	7
Staff, mean ± SD	7.5 ± 7.2
Teacher age, years (%, *n* = 152)	
18-25	16.3
26-40	43.3
41-55	27.9
> 55	12.5
Teacher education (%, *n* = 142)	
High School	23.2
Trade School, Some College or Associate Degree	52.8
Bachelor Degree	23.2
Graduate Degree	0.7
2-5 year old children enrolled, mean ± SD	22.9 ± 19.1
Racial/Ethnic distribution of 2–5 year old children	
(%, *n* = 327, estimated by program director)	
White/Caucasian	73.3
African American	8.9
Hispanic	7.6
American Indian	8.0
Multiple Race/Ethnicities	2.1

### Physical activity environment

The total physical activity environment and all sub-scales of the EPAO significantly improved for all sites combined (Table 3). The largest differences between baseline and 12 months were observed among sites with low supportive physical activity environment at baseline (*n* = 10, PA-EPAO score <11.2), specifically among the Physical Activity Equipment, Training/Education, and Policy sub-scales.

**Table 3:** Total Physical Activity Environment and Policy Assessment Observation (PA-EPAO) scores and sub-scales by all centers combined (*n* = 20) and by high (*n* = 10) and low (*n* = 10) scoring centers at baseline

EPAO component	Baseline	6-month	12-month	Difference score	p-value*
Total PA-EPAO	10.8 ± 1.9	15.5 ± 2.8	17.8 ± 2.0	7.0 ± 2.1	<0.01
High scoring centers	12.3 ± 0.9	16.4 ± 2.0	18.5 ± 0.8	6.2 ± 1.4	0.76
Low scoring centers	9.3 ± 1.3	14.5 ± 3.2	17.0 ± 2.6	7.7 ± 2.5	
PA sub-scales:					
Active opportunities	13.9 ± 3.3	17.2 ± 4.2	17.8 ± 3.5	3.9 ± 5.3	<0.01
High scoring centers	14.3 ± 3.9	18.7 ± 2.3	18.7 ± 2.3	4.4 ± 5.2	0.37
Low scoring centers	13.4 ± 2.9	15.8 ± 5.3	17.0 ± 4.3	3.4 ± 5.7	
Sedentary opportunities	10.5 ± 3.3	13.6 ± 3.4	18.7 ± 3.5	8.21 ± 3.5	<0.01
High scoring centers	10.9 ± 3.2	13.3 ± 0.0	19.3 ± 2.1	8.4 ± 2.8	0.18
Low scoring centers	9.9 ± 3.5	13.9 ± 4.9	18.0 ± 4.5	8.1 ± 3.5	
Sedentary environment	10.3 ± 5.9	14.3 ± 5.4	14.9 ± 4.8	4.7 ± 7.2	<0.01
High scoring centers	13.9 ± 3.8	15.9 ± 4.7	14.7 ± 5.3	0.7 ± 6.6	<0.01
Low scoring centers	6.6 ± 5.4	12.6 ± 5.8	15.3 ± 4.5	8.7 ± 5.5	
Portable play equipment	16.7 ± 3.3	18.3 ± 2.9	19.7 ± 1.3	3.0 ± 3.3	<0.01
High scoring centers	18.6 ± 2.4	18.9 ± 2.8	20.0 ± 0.0	1.4 ± 2.4	0.46
Low scoring centers	14.8 ± 2.9	17.7 ± 2.9	19.4 ± 1.8	4.6 ± 3.4	
Fixed play equipment	14.7 ± 4.1	17.9 ± 3.0	18.5 ± 2.2	3.8 ± 3.5	<0.001
High scoring centers	15.9 ± 3.5	18.1 ± 3.1	18.7 ± 1.7	2.7 ± 3.3	0.55
Low scoring centers	13.4 ± 4.4	17.8 ± 3.0	18.3 ± 2.6	4.8 ± 3.7	
Staff behaviors	16.0 ± 3.9	18.0 ± 4.4	19.4 ± 1.9	3.4 ± 3.7	<0.01
High scoring centers	17.6 ± 2.8	19.2 ± 1.7	20.0 ± 0.0	2.4 ± 4.4	0.33
Low scoring centers	14.4 ± 4.3	16.8 ± 5.9	18.8 ± 2.7	4.4 ± 4.4	
PA training & education	2.4 ± 3.8	14.6 ± 5.8	14.5 ± 6.5	12.2 ± 6.4	<0.01
High scoring centers	3.2 ± 4.7	16.3 ± 5.4	17.5 ± 3.5	14.3 ± 6.2	0.08
Low scoring centers	1.5 ± 2.4	13.0 ± 5.9	11.5 ± 7.5	10.0 ± 6.2	
PA policy	2.0 ± 4.1	9.6 ± 8.2	18.6 ± 4.6	16.6 ± 5.7	<0.01
High scoring centers	4.0 ± 5.2	11.0 ± 7.5	19.2 ± 1.9	15.2 ± 4.8	<0.71
Low scoring centers	0.0 ± 0.0	8.2 ± 9.0	18.0 ± 6.3	18.0 ± 6.3	

Data represent the mean ± standard deviation for each score. Significance was set at 0.05. *PA-EPAO* physical activity environment and policy assessment observation, *PA* physical activity

*p-values for each PA-EPAO sub-category (listed in bold) represent significant test for differences between baseline and 12-months calculated by t-test; p-values associated with high- (PA-EPAO ≥ 11.2) and low- (PA-EPAO < 11.2) scoring centers at baseline under each sub-category represent test of mean differences between those two groups by general linear modeling using Tukey’s adjustment for multiple comparisons

### Teacher-led physical activity and outside play time

Observed structured (i.e. teacher-led) physical activity (including both indoor and outdoor teacher-led activity) significantly increased from 30.9 ± 22.7 min at baseline to 82.3 ± 41.3 min at 12 months (Table 4, *p* < 0.01). Observed play time outside significantly increased from 84.5 ± 49.3 min at baseline to 107.8 ± 57.3 min at 6 months; however, 12-month scores were non significantly different from baseline. When analyzed by baseline PA-EPAO scores, the high-scoring centers increased outside time at 12 months (79.2 ± 61.9 to 92.0 ± 54.6 min) whereas the low PA-EPAO sites decreased outside time (89.7 ± 35.2 to 76.0 ± 39.9 min) resulting in a significant difference in the change scores over the intervention period (*p* < 0.01).

**Table 4:** Observed minutes of teacher-led^a^ physical activity and play time outside for all centers combined and by high and low scoring centers at baseline

Observed Time (min)	Baseline	6-month	12-month	Δ Baseline to 12-month	p-value*
Teacher-led PA	30.9 ± 22.7	76.8 ± 38.1	82.3 ± 40.7	51.3 ± 41.3	<0.01
High scoring centers	39.3 ± 21.9	93.9 ± 33.4	90.3 ± 36.4	51.0 ± 36.8	NS
Low scoring centers	22.6 ± 21.1	59.7 ± 35.9	74.2 ± 45.1	51.6 ± 47.3	
Outside time	84.5 ± 49.3	107.8 ± 57.3	84.2 ± 47.3	−0.25 ± 56.7	0.98
High scoring centers	79.2 ± 61.9	107.8 ± 56.7	92.0 ± 54.6	12.8 ± 72.6	<0.01
Low scoring centers	89.7 ± 35.2	107.7 ± 61.0	76.4 ± 39.9	−13.3 ± 33.3	

Data represent the mean ± standard deviation in minutes. Significance was set at 0.05. *PA* physical activity, *PA-EPAO* physical activity environment and policy assessment observation tool

^a^Teacher-led physical activity refers to structured activity. Outside play time refers to unstructured activity or activity that is not teacher initiated

*p-values for all centers combined for Teacher-led PA and outside play time (bold) represent test for differences between baseline and 12-month observations analyzed by t-test; p-values for high (PA-EPAO ≥ 11.2) and low (PA-EPAO < 11.2) centers at baseline represent mean differences between those two groups by PROC GLM using Tukey’s adjustment for multiple comparisons

### Child physical activity

At baseline, average percentage time spent in sedentary, light activity, and moderate-vigorous physical activity (MVPA), as measured by accelerometry, was 60.5 %, 37.2 % and 2.6 %, respectively (Table 5). Based on measurements of the same children who wore accelerometers at both baseline and at 12-months (*n* = 66), percent sedentary time decreased significantly over the 12-month intervention (−4.4 ± 14.2 %, *p* < 0.02), and percent MVPA time increased significantly (2.9 ± 3.0 %, *p* < 0.0001). This was associated with a decrease of 29.2 ± 2.6 min of sedentary time, and an increase of 12.0 ± 1.7 min MVPA. The difference in MVPA time was higher among sites with low baseline PA-EPAO vs. high PA-EPAO centers (3.6 %, 14.5 min vs. 2.0 %, 8.9 min). Furthermore, as structured (i.e. teacher led) activity increased, percent time sedentary decreased (*r* = −0.37, *p* < 0.05) and percent time in light physical activity increased (*r* = 0.35, *p* < 0.05) (data not shown). A positive trend for the correlation between teacher-led physical activity and percent time children were engaged in MVA was observed, but was not significant (*r* = 0.19, p >0.10).

**Table 5:** Child physical activity measured by accelerometry for all centers combined and by high and low scoring centers at baseline

	Baseline (*n* = 231)	6-month (*n* = 324)	12-month (*n* = 292)	Δ Baseline to 12-month (*n* = 66 matched pairs)^d^	p-value*
Total minutes measured	420.9 ± 114.6	381.3 ± 142.0	420.4 ± 113.4	−16.7 ± 127.0	
Sedentary, % time (minutes)^a^	60.5 ± 11.6 (254.6 ± 13.3)	59.4 ± 11.8 (226.5 ± 16.8)	60.0 ± 11.8 (252.2 ± 13.4)	−4.4 ± 14.6 (−29.2 ± 2.6)	0.02
High scoring centers^b^	59.0 ± 12.0 (248.3 ± 12.5)	58.7 ± 11.9 (223.8 ± 16.9)	58.9 ± 11.6 (247.6 ± 13.2)	−0.2 ± 13.5 (−4.2 ± 4.2)	0.04
Low scoring centers^c^	62.1 ± 10.9 (261.4 ± 11.4)	60.0 ± 11.7 (228.8 ± 16.6)	60.9 ± 11.9 256.0 ± 13.5)	−7.9 ± 14.8 (−49.4 ± 7.8)	
Light activity, % time (minutes)	37.2 ± 10.8 (156.6 ± 12.4)	37.4 ± 10.7 (142.6 ± 15.2)	36.5 ± 10.6 (153.4 ± 12.2)	1.5 ± 13.4 (−0.30 ± 3.4)	0.30
High scoring centers	38.3 ± 10.8 (161.2 ± 12.3)	37.9 ± 10.5 (144.5 ± 14.9)	37.6 ± 10.9 (158.1 ± 12.4)	−1.8 ± 13.4 (−10.6 ± 4.3)	0.08
Low scoring centers	36.0 ± 10.7 (151.5 ± 12.3)	36.9 ± 10.9 (129.7 ± 15.5)	35.4 ± 10.3 (148.8 ± 11.7)	4.3 ± 12.8 (7.6 ± 5.6)	
MVPA, % time (minutes)	2.3 ± 2.3 (9.6 ± 2.4)	3.3 ± 2.5 (12.6 ± 3.6)	3.5 ± 2.5 (14.7 ± 2.8)	2.9 ± 3.0 (12.0 ± 1.7)	<0.001
High scoring centers	2.6 ± 2.8 (10.9 ± 3.2)	3.4 ± 2.9 (12.9 ± 4.1)	3.5 ± 2.1 (14.7 ± 2.4)	2.0 ± 2.5 (8.9 ± 0.2)	0.04
Low scoring centers	1.90 ± 1.7 (8.0 ± 1.9)	3.2 ± 2.1 (12.2 ± 2.9)	3.6 ± 2.9 (15.1 ± 3.2)	3.6 ± 3.2 (14.5 ± 2.6)	

Data represent the mean ± standard deviation. Significance was set at 0.05. PA = physical activity; PA-EPAO = Physical Activity Environment and Policy Assessment Observation; MVPA = moderate to vigorous physical activity

^a^% time is calculated from total minutes of measured activity. Approximate minutes are reported in parentheses

^b^For high-scoring centers (PA-EPAO ≥ 11.2 at baseline), *n* = 120 at baseline, *n* = 148 at 6-month, *n* = 137 at 12-month

^c^For low-scoring centers (PA-EPAO < 11.2 at baseline), *n* = 111 at baseline, *n* = 176 at 6-month, *n* = 155 at 12-month

^d^Only children who were present for both the baseline and 12-month measurement were included in the Δ analysis; *n* = 66 for combined centers, *n* = 30 for high-scoring centers (PA-EPAO ≥ 11.2 at baseline), and *n* = 36 for low-scoring centers (PA-EPAO < 11.2 at baseline)

*p-values for total minutes and sedentary, light, and MVPA percentages represent differences between baseline and 12-month analyzed by t-test; p-values for high (PA-EPAO ≥ 11.2) and low (PA-EPAO < 11.2) centers at baseline represent mean differences between those two groups by PROC GLM using Tukey’s adjustment for multiple comparisons

### Physical activity policy

At the 12-month time point, 19 out of 20 sites had written policies on physical activity regarding active play time (structured teacher-led and unstructured play) compared to one site at baseline. The one site that did not have a written policy at the end of the intervention was undergoing policy review with state headquarters. Evidence-based policy changes included statements regarding the following: the amount of active play provided each day, the amount of teacher-led active play, the amount of time children spend outdoors each day, appropriate clothing for active play, not taking away active play as a punishment, providing active play as reward, the type of indoor and outdoor play equipment available, staff behavior and supervision during outdoor play, and the number and frequency of physical activity education sessions provided to children, staff, and parents. As shown in Table 3, mean EPAO score (on a scale of 0–20) significantly improved at 12 months for Physical Activity Training and Education (14.5 ± 6.5 vs. 2.4 ± 3.8 at baseline, *p* < 0.01) and for Physical Activity Policy (18.6 ± 4.6 vs. 2.0 ± 4.1 at baseline, *p* < 0.01).

## Discussion

The Active Early intervention was designed to provide ECE programs with the resources and tools (i.e., the Active Early guide) to offer 120 min of physical activity daily, with half of that being structured (i.e. teacher-led) activity. This study demonstrated that through policy changes, training, and provision of resources, 60 min of teacher-led activity was achieved, resulting in an average of 168 total minutes of physical activity among children wearing accelerometers. The physical activity environment and behaviors improved after administration of the Active Early intervention, which included supportive training and resources to implement physical activity policies and best practices. The improvements over the intervention period were observed to be greatest for centers that initially scored lower at baseline for these measures of physical activity environment and behaviors. Moreover, these changes were demonstrated across family, small group, and large group centers, suggesting the Active Early protocol was effective for a variety of ECE settings. This study adds to accumulating data [25] regarding the implementation of a physical activity intervention in ECE settings by assessing both the physical activity environment as well as child-level activity changes as result of a policy-level intervention. Similar to Pfiefer et al. [25], this intervention showed that by providing resources, intensive training and support to child care providers, opportunities for physical activity can increase in the child care environment resulting in increased active time among children.

Increased awareness of the importance of the early childhood period has prompted many local and state organizations to examine regulations in ECE settings. A recent report suggested that while most states had written regulations addressing physical activity, few states had regulations addressing 3 or more of the 5 key IOM recommendations related to physical activity in childcare [26]. Moreover, significant attention has been given to the identification and implementation of best practices in ECE settings [27], but few studies have evaluated outcomes related to best practices or other interventions in child care centers. Some studies have examined compliance with such regulations and best practices [28], while other groups have examined isolated components of behavior, such as provider perceptions [29] or modeling of healthy behaviors [30], to determine effects on early child health. In contrast, our approach utilizing the Active Early guide along with training and micro-grant support included a comprehensive assessment of behavior, environmental factors, and policies supporting increased physical activity in ECE. Strong state-level regulations and best practices are critical components of obesity prevention efforts, but outcome-focused analysis of interventions designed to support implementation of these approaches, such as the Active Early guide described here, are necessary to support increases in physical activity levels in early childhood.

In Wisconsin, these data have supported state-wide training and policy measures for physical activity in the ECE setting. As a result of this study, over 71 trainings on the Active Early guide have been conducted, reaching over 1,632 providers statewide; 10,000 copies of the Active Early guide in English and 1,000 copies in Spanish are currently in circulation. In Wisconsin, physical activity was integrated into the statewide voluntary quality improvement rating system, YoungStar, as part of an optional “wellness point”. To achieve the physical activity component of this “wellness point” (which also includes a nutrition component), programs must integrate a minimum of 60 min of physical activity into their daily schedule. Although this physical activity point remains optional and falls short of the 120 min recommended for this age group, the recognition of physical activity in the Wisconsin quality rating and improvement system is significant. In the first year of YoungStar (January 2011 to January 2012), 504 providers earned the optional physical activity point.

There are several limitations of our approach to note. First, because this project was funded as a public health evaluation study, this study utilized a quasi-experimental study design that lacked a control group, only allowing us to assess changes over the course of the 12-month intervention. Therefore, we are unable to account for other changes and regulations statewide that may have impacted the physical activity environment and behaviors in the centers included in this analysis. Another limitation was that the selection of sites was not random, and observation dates were pre-arranged with providers rather than unannounced. Provider and staff awareness of evaluation times may have biased results more favorably. While the Active Early guide is currently publicly available for use, centers choosing to utilize this resource would not have access to the microgrants provided as part of this analysis. Microgrant purchases made during the study period included gross motor, music, and movement equipment; children’s books to promote physical activity; space modification to allow for more indoor activity; staff training; and curriculum or other resources. Because the centers included in this study were also modestly incentivized to purchase equipment and other resources to improve physical activity, some of the measures may be related to equipment availability. Although we observed that centers initially scoring low on baseline PA-EPAO demonstrated greater improvement than those scoring higher at baseline, these results may have been the result of regression to the mean. Finally, the same children were not always present on measurement/observation days. To address this issue, accelerometry data comparing pre-post differences only included children present for both measurements, although this limited the overall sample size for the physical activity variables.

## Conclusions

Implementing the Active Early protocol has shown that offering 120 min of physical activity each day and achieving at least 60 min of teacher-led activity in ECE settings is feasible. From preliminary review of observations, it appears that the strategies to meet this objective need to be focused on teacher-led physical activity time. As recommended in the Active Early guide, opportunities for physical activity are highly increased with active transition times during the day by integrating gross motor and/or music and movement. Additionally, a major factor in the success of Active Early was the training and technical assistance offered to ECE professionals. The sustainability of physical activity policies most likely depends on those organizations that support and train ECE providers and for states to establish/set higher standards for physical activity policies and incentives in regulated child care.

## Abbreviations

BMI, body mass index; CPPW, communities putting prevention to work; ECE, early care and education; EPAO, environment and policy assessment and observation tool; MET, metabolic equivalent; MVPA, moderate to vigorous physical activity; NAP SACC, nutrition and physical activity self-assessment for child care; NPAO, Wisconsin Department of Health Services’ Nutrition, Physical Activity, and Obesity Program; WECOPI, Wisconsin Early Childhood Obesity Prevention Initiative; WI, Wisconsin
